# cMET in NSCLC: Can We Cut off the Head of the Hydra? From the Pathway to the Resistance

**DOI:** 10.3390/cancers7020556

**Published:** 2015-03-25

**Authors:** Nele Van Der Steen, Patrick Pauwels, Ignacio Gil-Bazo, Eduardo Castañon, Luis Raez, Federico Cappuzzo, Christian Rolfo

**Affiliations:** 1 Center for Oncological Research Antwerp, University of Antwerp, Universiteitsplein 1, Wilrijk 2610, Belgium; E-Mails: Nele.VanDerSteen@uantwerpen.be (N.V.D.S.); Patrick.Pauwels@uza.be (P.P.); 2 Molecular Pathology Unit, Pathology Department, Antwerp University Hospital, Wilrijkstraat 10, Edegem 2650, Belgium; 3 Department of Oncology, Clínica Universidad de Navarra, Pamplona 31008, Spain; E-Mails: igbazo@unav.es (I.G.-B.); ecastanon@unav.es (E.C.); 4 Thoracic Oncology Program, Memorial Cancer Institute, Memorial Health Care System, Pembroke Pines, FL 33024, USA; E-Mail: lraez@mhs.net; 5 Medical Oncology Department, Istituto Toscano Tumori, Ospedale Civile, Livorno, Italy viale Alfieri 36, Livorno 57100, Italy; E-Mail: f.cappuzzo@gmail.com; 6 Phase I-Early Clinical Trials Unit, Oncology Department, Antwerp University Hospital, Wilrijkstraat 10, Edegem 2650, Belgium

**Keywords:** cMET, NSCLC, targeted therapies

## Abstract

In the last decade, the tyrosine kinase receptor cMET, together with its ligand hepatocyte growth factor (HGF), has become a target in non-small cell lung cancer (NSCLC). Signalization via cMET stimulates several oncological processes amongst which are cell motility, invasion and metastasis. It also confers resistance against several currently used targeted therapies, e.g., epidermal growth factor receptor (EGFR) inhibitors. In this review, we will discuss the basic structure of cMET and the most important signaling pathways. We will also look into aberrations in the signaling and the effects thereof in cancer growth, with the focus on NSCLC. Finally, we will discuss the role of cMET as resistance mechanism.

## 1. Introduction

Nowadays, a lot of attention is going to the cMET receptor, given its importance as a resistance mechanism for targeted therapies but also as a codriver of tumor growth and metastasis. Besides its fame for its involvement in cancer growth, cMET also plays an important role during embryonic development and in wound healing [1,2]. Given the name for its ligand HGF, hepatocyte growth factor, cMET signaling is an important mitogen for liver cells [3]. The cMET pathway is also necessary for the forming of tubules [4], as is the case for the development of blood vessels [5], lymph vessels [6] and for the correct development of the placenta [7] and kidney [8]. Finally, in adults, it has an important function in wound healing, where it stimulates cell migration. In this review we want to discuss in detail the structure of cMET and HGF, describe the pathway into detail and finally we will discuss the resistance mechanisms used with a focus on NSCLC.

## 2. cMET and HGF Structure

The cMET and HGF genes are both located at chromosome 7, at locus 7q31 (GeneID 4233) and 7q21.1 (GeneID 3082) respectively. cMET gene transcription and translation results in a precursor protein. Cleavage by furin between R_307_ and S_308_ leads to the formation of an α- and β-chain that are linked by disulphide bonds resulting in a mature cMET protein [9,10]. The α-chain is situated extracellular, whereas the β-chain contains an extracellular, membrane-spanning and intracellular part [11]. The extracellular sema-domain contains the ligand binding domain, that is build up as a 7-bladed β-propeller [12]. The C-terminal region of the β-chain contains the regulatory juxtamembrane domain and the docking sites for the adaptor proteins (see cMET pathway) [13]. The mature receptor is expressed by several different cell types like epithelial cells, endothelial cells, mesenchymal stem cells and neurons [14].

The main sources for HGF are mesenchymal cells [15]. HGF, like cMET, is translated as single-chained pro-HGF and can be cleaved by several proteins like uPA (urokinase plasminogen activator), tPA (tissue plasminogen activator) and coagulation factors amongst others [16,17]. This cleaving results in mature HGF with an α- and β-chain, linked by disulphide bonds. This mature HGF is bound by heparin sulphate glycosaminoglycanes, which is necessary for ligand oligomerisation, cMET binding and activation. This sequestering of HGF finally limits its diffusion range [18,19].

## 3. The cMET Pathway Disassembled

### 3.1. Ligand-Dependent Activation

The mature ligand HGF binds the receptor, which leads to receptor dimerization and phosphorylation of Tyr1234 and Tyr1235. This ultimately results in the phosphorylation of the intracellular docking sites Tyr1349 and Tyr1356. At these sites, the adaptor proteins Grb2 (growth factor receptor bound protein 2), Gab1 (Grb2 associated binding protein 1) and SHC (Src homology 2 domain containing) will bind to the receptor, thus activating downstream signaling [13,20,21].

#### 3.1.1. MAPK Cascades

There are two main MAPK (mitogen activated protein kinase) cascades that are activated by cMET: MEK/ERK and MEK/JNK, both through the activation of Ras.

Here, the cMET receptor relies on CD44v6 as coreceptor and HGF-binding for activation [22]. The cytoplasmic domain of CD44v6 associates the receptor with the cytoskeleton and is required for recruitment of the ERM complex (Ezrin-Radixin-Moesin), which is needed for Ras activation [23]. Ras directly activates the MEK/ERK pathway and the MEK/JNK pathway, resulting in cell proliferation and transformation. Besides direct activation of Ras, also the deactivation of the Ras-inhibitor p120-Ras-GAP is provoked by cMET signaling, thus enhancing MAPK signaling [13,24,25].

#### 3.1.2. PI3K-Akt

The second main signaling pathway of cMET is the PI3K-Akt (phosphoinositide-3-kinase/protein kinase B) pathway. PI3K is either directly activated by cMET or by activation of Ras [26]. Signaling through PI3K-Akt leads to different processes that are involved in cell growth, proliferation and escape to apoptosis. Firstly, it leads to mTOR activation, which initiates several processes involved in protein translation and cell growth [27]. Secondly, by inhibiting GSK3β (glycogen synthase kinase 3β) PI3K leads to cell proliferation [28]. Thirdly, PI3K-Akt signaling leads to protection against apoptosis by inhibition of BAD (BCL2 antagonist of cell death) [29] and the activation of MDM2 (mouse double minute 2) which in turn leads to p53 degradation. Besides directly, MDM2 also indirectly gets activated by mTOR [30]. Finally, Akt phosphorylates procaspase 9, which leads to the inhibition of its protease activity and protection from apoptosis [31].

#### 3.1.3. STAT3

Upon activation of cMET, STAT3 (signal transducer and activator of transcription 3) associates with the receptor and gets phosphorylated [32]. Next, the phosphorylated STAT3 dissociates from the receptor and homodimerizes. Finally, it translocates to the nucleus and there, serves as a transcription factor for several genes involved in cell proliferation and differentiation (reviewed in [33]).

#### 3.1.4. NF-κB

Another transcription factor that is involved in cMET signaling is NF-κB (Nuclear Factor-κB). Hereby IKK (Inhibitor of NF-κB kinase) is activated either through Src or PI3K-Akt, where after it phosphorylates IκB (Inhibitor of NF-κB). Normally NF-κB is sequestered by its inhibitor in the cytoplasm. Upon IκB phosphorylation, NF-κB is released and translocates to the nucleus were it functions as a transcription factor for several genes that are involved in tubulogenesis [34], mitogenesis and are anti-apoptotic [35].

### 3.2. Ligand Independent Activation

#### 3.2.1. α5β1-Integrines-FAK

Besides activation through the binding of HGF, cell adhesion can be responsible for the activation of cMET [36,37]. After the binding of α5β1-integrines to fibronectin or type IV collagen, this complex associates with cMET, thus activating the autophosphorylation of the receptor [38]. Once cMET is activated, it associates and phosphorylates Src, which in turn activates FAK (focal adhesion kinase) [39]. The many ways in which FAK is involved in cancer growth are beyond the scope of this paper, but have been recently reviewed by Sulzmaier *et al.* [40].

#### 3.2.2. Sema4D

Sema4D and its receptor PlexinB1 are mostly known for their involvement in axonal guidance [41]. However, in recent years their role in cancer growth and angiogenesis is being revealed. It has become clear that binding of Sema4D on cells expressing both cMET and PlexinB1, induces cMET clustering and activation, and triggers invasive growth and angiogenesis [42]. When looking specifically at the expression of Sema4D in tumor samples, this was found to be elevated in several tumor types like HNSCC (head and neck squamous cell carcinoma), prostate, colon, breast and lung cancer [43]. Conrotto *et al.*, on the contrary, have selected cell lines of colon, liver, pancreas and gastric cancer that overexpressed PlexinB1 and found that PlexinB1 was phosphorylated and associated with cMET. By downregulating either PlexinB1 or cMET through RNA interference, they were able to decrease the phosphorylation level of cMET and PlexinB1 [44]. It should be noted that Sema4D and PlexinB1 do not always appear to have a tumor-promoting effect, but can also function as tumor-suppressors. However, this seems to differ between tumor type with a tumor-suppressor effect mainly noted in ER-positive breast cancer and melanoma, were binding of Sema4D to PlexinB1 leads to a decrease in cMET phosphorylation [45,46,47].

### 3.3. cMET Internalization

After the binding of HGF, the activated cMET receptor is ubiquitinised by the binding of c-Cbl (Casitas B-lineage lymphoma) at phospho-Tyr1003 in the juxtamembrane domain [48], after which it is quickly internalized through the invagination of clathrin coated pits [49] in endosomes. Next, the receptor can either be recycled back to the plasmamembrane by GGA3 (Golgi-associated, gamma adaptin ear containing, ARF binding protein 3) [50], or be degraded by the lysosomal pathways [51]. During transport across the different endosomes, cMET continues to signal, which evokes different reactions than signaling from the cell membrane. As such, it has been shown that signaling of cMET in peripheral endosomes is necessary for the full activation of ERK1/2 [52]. Furthermore, the changing cellular location also plays a role, as is shown for STAT3. Here perinuclear signaling of cMET is necessary to phosphorylate STAT3 close to the nucleus, so as to overcome the intrinsic weakness of this signal [53]. A third example of endosomal signaling of cMET is Rac1. Once internalized in peripheral endosomes, cMET activates Rac1, which in turn translocates to the plasmamembrane and here changes cytoskeletal dynamics, causing membrane ruffling and thus plays a role in cell migration [54].

### 3.4. cMET Shedding

Besides cMET degradation following ligand stimulation, there is also the ligand-independent cMET shedding. This process occurs at a basal level in epithelial cells, but can also be stimulated by e.g., antibodies against cMET [55]. The shedding process is composed of several steps. First, the receptor is cleaved by metalloproteases like ADAM10 [56] or ADAM17 [55] (a disintegrin and metalloprotease), which results in an extracellular and intracellular cMET fragment. The extracellular fragment is still able to bind HGF, functioning as a decoy receptor. Next, the membrane-bound intracellular part is cleaved in a γ-secretase/presenilin dependent manner. These two intracellular parts are highly unstable and are degraded by the 26S proteasome [55].

## 4. Aberrant cMET Signaling

### 4.1. cMET Mutations

**Figure 1:**
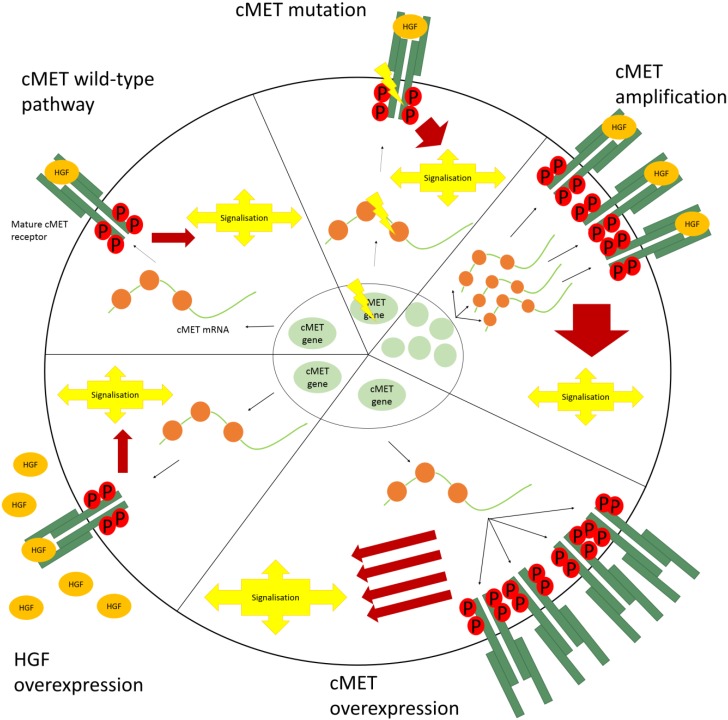
Schematic representation of aberrant cMET signaling.

There are several domains of the cMET receptor that play an important role in the regulation/activation of the receptor, and a mutation in each of these domains has the opportunity to deregulate the receptor activation and thus have oncogenic potential (Figure 1). A first domain is the extracellular HGF-binding domain. Until now there are two mutations discovered in this domain that influence the affinity for HGF, with the E168D mutation conferring higher affinity for HGF and the N375S mutation showing lower affinity [57]. Next, the juxtamembrane domain contains an autoinhibition loop to block the autophosphorylation of the receptor in the absence of ligand-binding. Here the R988C and T1010I mutations have been described, although there are contradicting reports in literature about their tumorigenicity [58,59,60,61]. Besides point mutations, there are also several splice site mutations known that effect exon 14, the juxtamembrane domain. Here at least a part of the regulatory loop is spliced out of the final protein, causing aberrant signaling [62]. A third important domain is the tyrosine kinase domain. Here there are mutations that result in a higher activity of cMET, e.g., Y1230C/H and D1228H [63] or are able to transform fibroblasts [64]. Another possible effect is a change in sensitivity for cMET small molecule inhibitors [63]. Finally these mutations can also stabilize the active conformation of the receptor (L1213V and M1268T) [65] or lower the activation threshold (M1250T and D1228H) [66]. The above described mutations are picked up in several tumor types, amongst which are NSCLC and SCLC, gastric carcinoma and hereditary papillary renal cell carcinoma. However, the exact prevalence of these mutations in NSCLC is not known.

### 4.2. Amplification

Amplification of the *cMET* gene is another mechanism to disturb cMET signaling (Figure 1). Nowadays, there is no clear cut-off value to determine amplification, nor is there a real consensus about the way to test this (PCR-based or by *in situ* hybridization). It is also necessary to make a distinction between the past treatments of the tested patients, resulting in primary or post-treatment amplification. For primary amplification, the percentages in literature vary around 3% to 4% [67,68], whereas for patients treated with erlotinib/gefitinib this percentage is ranging between 15% and 25% [67,68,69].

### 4.3. Overexpression

A third possibility for a disturbed cMET signaling is the overexpression of cMET, with or without amplification (Figure 1). The percentages of NSCLC tumors with cMET overexpression vary largely amongst the different studies, and range between 15% and 60% [70,71,72,73]. This overexpression can be the result of changes at the genetic level, the transcriptional or the translational level. At the genetic level, gene amplification can result in a higher transcriptional activity and thus more protein production [74]. Given the fact that overexpression is not always accompanied by gene amplification, modifications at the transcriptional level are also possible, e.g., higher promotor activity by epigenetic or histone modifications [75]. Next, the mRNA can be translated at a higher speed by the ribosomes or miRNAs involved in the control of cMET [76]. However, which of these mechanisms forms the basis of cMET overexpression, and whether it can explain all overexpressing cases remains to be discovered.

### 4.4. HGF Overexpression

Besides changes at the receptor level, also the ligand HGF can influence cMET signaling (Figure 1). Under normal conditions, HGF is mainly produced by stromal cells. However, it is also possible that the tumor cells themselves produce HGF, enabling cMET signaling in an autocrine way [77]. When looking at HGF expression, it is important to distinguish between autocrine signaling (HGF expression in the tumor cells) and paracrine signaling (HGF expression in stromal cells). For the expression on tumor cells the numbers vary between 25% and 83% [78,79,80], and for stromal expression the percentages are between 3% and 20%.

## 5. cMET as a Resistance Mechanism in the Treatment of NSCLC

### 5.1. cMET and Ionizing Radiation

In the past few years, several reports have been published about the upregulation of cMET after ionizing radiation therapy (IR) [81], with *in vitro* assays showing that cMET amplification increases in a dose-dependent way [82]. De Bacco *et al.* found a causal role for IR in the upregulation of cMET, with cMET induction starting at doses between 1 and 5 Gray and reaching a plateau at doses between 5 and 10 Gray [83]. This upregulation can be the result of different reactions of the cells on therapy. A first reaction is the “stress-and-recovery” response of the cells [84], with NF-κB and ATM (Ataxia telangiectasia mutated) upregulating cMET expression [83]. Another explanation can be that after IR, cell growth and epithelial-mesenchymal-transition is needed for the tissues to repair the induced damage, in which cMET plays an important role [85]. Since IR causes double stranded DNA breaks [86], a third possibility for the upregulation of cMET is its involvement in homologues recombination mediated DNA-repair, more specifically in the assembling of the BRCA1-Rad51 complex [87]. Finally it has been shown that IR can stimulate HGF secretion in glioblastoma [88]. Whether or not this is also the case for NSCLC remains to be investigated. However, despite the many different roles of cMET in the cellular response after IR, the discussion whether or not cMET upregulation leads to more metastases in irradiated patients remains open.

### 5.2. cMET and Chemotherapy

The HGF-cMET axis also plays a role in chemoresistance. Firstly, since activation of cMET contributes to the stem cell character of tumor cells, it contributes to the chemoresistance of these cells (reviewed in [89]). Secondly, it has been shown that overexpression and/or activation of cMET contributes to resistance against gemcitabine, cisplatin and paclitaxel [90,91]. Tang *et al.* have discovered that this resistance is dependent on cMET signaling through the PI3K/Akt and ERK pathways [91], were Chen *et al.* discovered a dependency on FAK [92]. Lastly, by combining a cMET inhibitor with chemotherapeutics (e.g., carboplatin, paclitaxel) chemoresistance could be reversed [93,94]. However, this was not the case in all tested chemotherapeutics as was shown by Yashiro *et al.* [94]. The question if cMET amplification plays an important role in chemoresistance, however, remains unsolved.

### 5.3. cMET and Hypoxia

Even in lung tumors, hypoxic regions can arise, caused by the malfunction of aberrantly formed blood vessels or when rapidly growing tumor cells outgrow their oxygen supply. In hypoxic regions it has been shown that cMET and HGF are both transcriptionally upregulated [95], causing overexpression of the receptor and its ligand in hypoxic regions [96]. This upregulation can be explained by the tendency of cells to escape the hypoxic environment, by means of epithelial-mesenchymal transition. Besides cMET, also an upregulation of MMP (matrix metalloproteases) can be seen, which shows increased activity under cMET signaling, thus promoting cell invasion [95]. Since some therapies aim at the inhibition of the formation of new blood vessels (e.g., VEGFR inhibitors) or need oxygen to fully employ their toxicity (e.g., some chemotherapeutics or radiotherapy), the upregulation of cMET and HGF under hypoxic conditions should be taken into account.

### 5.4. cMET and EGFR-Inhibition

The epidermal growth factor receptor (EGFR) is a known oncogenic driver in NSCLC. Sensitizing mutations in EGFR are good biomarkers for response to EGFR-inhibitors (e.g., erlotinib, gefitinib) [97,98]. However, most patients relapse from this treatment and become resistant against these inhibitors, or do not respond at all due to primary resistance. This resistance can be caused by a T790M resistance mutation in EGFR [98] or the expression/activation of cMET, AXL or other pathways [99]. In the case of cMET, both activation/phosphorylation [99] or amplification [69,100] are known to confer primary or secondary resistance against EGFR-inhibitors. In the *in vitro* study by Engelman *et al.*, it has been found that EGFR-TKI resistant cell lines showed cMET-amplification, and by inhibiting cMET expression through shRNA the sensitivity to EGFR-TKIs was restored [69]. When looking at the percentages of cMET amplification, Cappuzzo *et al.* have shown that the amount of patients with cMET amplification was lower in EGFR-TKI naïve patients, as compared to patients with acquired resistance against EGFR-TKIs [101]. As such, dual inhibition of both EGFR and cMET may be able to overcome this resistance. Although the synergistic effect of EGFR and cMET inhibitors has been shown *in vitro* [102,103,104], the beneficial effect of these combinations remains to be shown in patients.

## 6. Discussion

In the last decade the cMET receptor has become more and more important in NSCLC, mainly as a resistance mechanism against existing targeted therapies but also as a primary target. In the study by Huang *et al.*, cMET amplification conferred a poor prognosis in EGFR-wild type patients, which leads to the question whether cMET inhibitors may be used as an early treatment in this population [105]. The increasing importance of cMET is illustrated by the increasing amount of cMET inhibitors in clinical trials, examples of which are Onartuzumab, Ficlatuzumab and Rilotumumab as antibody therapies and Crizotinib, Foretinib and Golvatinib as small molecule inhibitors. Some studies even aim for double inhibition of both the EGFR and cMET pathway.

However, the current development and FDA approval of cMET inhibitors is not going smoothly. One example of this is Rilotumumab, were phase III trials in cMET overexpressing gastroesophageal patients, combining Rilotumumab with chemotherapy, have been stopped recently due to increased death rate in the low cMET expressing group. For a possible explanation we could refer to Yashiro *et al.* were it was shown that combination therapy of the cMET inhibitor SU11274 with cisplatin worked synergistically, but combination with other chemotherapeutics like 5-fluoro-uracil and gemcitabine worked antagonistically. However, recent results in several clinical trials have been disappointing, raising the question which biomarker should be used for patient selection. Is it representative to determine the level of cMET expression by immunohistochemistry or is it necessary to directly look at gene amplification by FISH (fluorescent *in situ* hybridization)? Dziadziuszko *et al.* performed a correlation analysis of the cMET gene copy number by SISH (silver *in situ* hybridization) and protein expression by immunohistochemistry. Amongst 138 patients, they observed a moderate correlation (Pearson’s *r* = 0.4, *p* < 0.001) with these techniques [106]. Although this result is statistically significant, hybridization is a more accurate technique to detect gene amplification, whereas IHC may be biased since it does not distinguishes amplification from overexpression. Another problem arises when taking hybridization techniques as the gold standard, namely is it enough to select patients with a ratio between the centromere and the gene of just over 2, or should we only select those samples that show high amplification (ratio of 5 and more)? This decision is even more important in order to select those patients with the greatest benefit to be enrolled in clinical trials with cMET inhibitors. Even more so, with a lack of consensus it will become very difficult to compare data coming from trials in which IHC or FISH have been used according to different criteria. Finally, cMET expression and/or amplification may also evolve with (previous) therapies, leading to the question if pretreatment biopsies should be required. However, given the fact that cMET expression may vary within different regions of the primary tumor and, moreover, can be enriched in metastasis [99] it remains to be seen if a biopsy can be representative for the whole tumor burden of a patient.

Finally, as cMET inhibition is in the spotlight, mechanisms of resistance against cMET inhibitors are being discovered, bridging to other pathways such as Wnt or mTOR [107], with presumably more connections on the way.

## 7. Conclusions

By describing the structure and the different signaling routes of cMET, we aimed to provide insight in the different roles of cMET in tumor growth and the possible strategies and implication of cMET inhibition, with the focus on NSCLC, and highlight the remaining questions for further research.

Can we cut off the head of the Hydra? Unfortunately, we have not yet succeeded in preventing new heads from growing, but maybe the finding of a “golden” biomarker can be the sword we need to conquer cMET.

## References

[1] Achim C.L., Katyal S., Wiley C., Shiratori M., Wang G., Oshika E., Petersen B.E., Li J.M., Michalopoulos G.K. (1997). Expression of HGF and cMET in the developing and adult brain. Dev. Brain Res..

[2] Conway K.P., Price P.E., Harding K.G., Jiang W.G. (2006). The molecular and clinical impact of hepatocyte growth factor, its receptor, activators, and inhibitors in wound healing. Wound Repair Regen..

[3] Borowiak M., Garratt A.N., Wüstefeld T., Strehle M., Trautwein C., Birchmeier C. (2004). Met provides essential signals for liver regeneration. Proc. Natl. Acad. Sci. USA.

[4] Montesano R., Soriano J.V., Malinda K.M., Ponce M.L., Bafico A., Kleinman H.K., Bottaro D.P., Aaronson S. (1998). A Differential effects of hepatocyte growth factor isoforms on epithelial and endothelial tubulogenesis. Cell Growth Differ..

[5] Ding S., Merkulova-rainon T., Han Z.C. (2003). HGF receptor up-regulation contributes to the angiogenic phenotype of human endothelial cells and promotes angiogenesis *in vitro*. Blood.

[6] Kajiya K., Hirakawa S., Ma B., Drinnenberg I., Detmar M. (2005). Hepatocyte growth factor promotes lymphatic vessel formation and function. EMBO J..

[7] Stewart F. (1996). Roles of mesenchymal-epithelial interactions and hepatocyte growth factor-scatter factor (HGF-SF) in placental development. Rev. Reprod..

[8] Santos O., Barros E., Yang X.-M., Matsumoto K., Nakamura T., Park M., Nigam S. (1994). Involvement of hepatocyte growth factor in kidney development. Dev. Biol..

[9] Komada M., Hatsuzawa K., Shibamoto S., Ito F., Nakayama K., Kitamura N. (1993). Proteolytic processing of the hepatocyte growth factor/scatter factor receptor by furin. FEBS Lett..

[10] Tempest P., Stratton M., Cooper C. (1988). Structure of the met protein and variation of met protein kinase activity among human tumour cell lines. Br. J. Cancer.

[11] Giordano S., Flavia M., Renzo D.I., Ferracini R., Chiado-piat L., Comoglio P.M. (1988). p145, a protein with associated tyrosine kinase activity in human gastric carcinoma cell line. Mol. Cell Biol..

[12] Gherardi E., Youles M.E., Miguel R.N., Blundell T.L., Iamele L., Gough J., Bandyopadhyay A., Hartmann G., Butler P.J.G. (2003). Functional map and domain structure of MET, the product of the c-met protooncogene and receptor for hepatocyte growth factor/scatter factor. Proc. Natl. Acad. Sci. USA.

[13] Ponzetto C., Giordano S., Graxiani A., Panayotou G., Comoglio P.M.Y., Bardelli A., Zhen Z., Maina F., dalla Zonca P., Giordano S. (1994). A multifunctional docking site mediates signaling and transformation by the hepatocyte growth factor/scatter factor receptor family. Cell.

[14] Jung W., Castren E., Odenthal M., vande Woude G.F., Ishii T., Dienes H.P., Lindholm D., Schirmacher P. (1994). Expression and functional interaction of hepatocyte growth factor—Scatter factor and its receptor. J. Cell Biol..

[15] Sonnenberg E., Meyer D., Weidner K.M., Birchmeier C. (1993). Scatter factor/hepatocyte growth factor and its receptor, the c-met tyrosine kinase, can mediate a signal exchange between mesenchyme and epithelia during mouse development. J. Cell Biol..

[16] Mars W.M., Zarnegar R., Michalopoulos G.K. (1993). Activation of hepatocyte growth factor by the plasminogen activators uPA and tPA. Am. J. Pathol..

[17] Owen K.A., Qiu D., Alves J., Schumacher A.M., Kilpatrick L.M., Li J., Harris J.L., Ellis V. (2010). Pericellular activation of hepatocyte growth factor by the transmembrane serine proteases matriptase and hepsin, but not by the membrane-associated protease uPA. Biochem. J..

[18] Mizuno K., Inoue H., Hagiya M., Shimizu S., Nose T., Shimohigasho Y., Nakamura T. (1994). Hairpin loop and second kringle domain are essential sites heparin binding and biological activity of hepatocyte growth factor. J. Biol. Chem..

[19] Sakata H., Stahl S.J., Taylor W.G., Rosenberg J.M., Sakaguchi K., Wingfield P.T., Rubin J.S. (1997). Heparin binding and oligomerization of hepatocyte growth factor/scatter factor isoforms. Heparan Sulfate glycosaminoglycan requirement for met binding and signaling. J. Biol. Chem..

[20] Fixman E., Fournier T., Kamikura D., Naujokas M., Park M. (1996). Pathways downstream of Shc and Grb2 are required for cell transformation by the Tpr-Met oncoprotein. J. Biol. Chem..

[21] Weinder K.M., di Cesare S., Sachs M., Brinkmann V., Behrens J., Birchmeier W. (1996). Interaction between Gab1 and the c-Met receptor tyrosine kinase is responsible for epithelial morphogenesis. Nature.

[22] Orian-rousseau V., Chen L., Sleeman J.P., Herrlich P., Ponta H. (2002). CD44 is required for two consecutive steps in HGF/c-Met signaling. Genes Dev..

[23] Orian-Rousseau V., Morrison H., Matzke A., Kastilan T., Pace G., Herrlich P., Ponta H. (2007). Hepatocyte growth factor-induced Ras activation requires ERM proteins linked to both CD44v6 and F-Actin. Mol. Biol. Cell.

[24] Montagner A., Yart A., Dance M., Perret B., Salles J.-P., Raynal P. (2005). A novel role for Gab1 and SHP2 in epidermal growth factor-induced Ras activation. J. Biol. Chem..

[25] Graziani A., Gramaglia D., dalla Zonca P., Comoglio P.M. (1993). Hepatocyte growth factor/scatter factor stimulates the Ras-guanine nucleotide exchanger. J. Biol. Chem..

[26] Xiao G.H., Jeffers M., Bellacosa A., Mitsuuchi Y., vande Woude G.F., Testa J.R. (2001). Anti-apoptotic signaling by hepatocyte growth factor/Met via the phosphatidylinositol 3-kinase/Akt and mitogen-activated protein kinase pathways. Proc. Natl. Acad. Sci. USA.

[27] Zoncu R., Sabatini D., Efeyan A. (2012). mTOR: From growth signal integration to cancer, diabetes and ageing. Nat. Rev. Mol. Cell Biol..

[28] Hoeflich K.P., Luo J., Rubie E.A., Tsao M., Jin O., Woodgett J.R. (2000). Requirement for glycogen synthase kinase-3β in cell survival and NF-kB activation. Nature.

[29] Del Peso L., Gonzalez-Garcia M., Page C., Herrera R., Nunez G. (1997). Interleukin-3-Induced phosphorylation of BAD through the protein kinase Akt. Science.

[30] Moumen A., Patané S., Porras A., Dono R., Maina F. (2007). Met acts on Mdm2 via mTOR to signal cell survival during development. Development.

[31] Cardone M.H. (1998). Regulation of cell death protease caspase-9 by phosphorylation. Science.

[32] Zhang Y.-W., Wang L.-M., Jove R., vande Woude G.F. (2002). Requirement of Stat3 signaling for HGF/SF-Met mediated tumorigenesis. Oncogene.

[33] Yu H., Pardoll D., Jove R. (2009). STATs in cancer inflammation and immunity: A leading role for STAT3. Nat. Rev. Cancer.

[34] Müller M., Morotti A., Ponzetto C. (2002). Activation of NF-kB is essential for hepatocyte growth factor-mediated proliferation and tubulogenesis. Mol. Cell. Biol..

[35] Fan S., Gao M., Meng Q., Laterra J.J., Symons M.H., Coniglio S., Pestell R.G., Goldberg I.D., Rosen E.M. (2005). Role of NF-kappaB signaling in hepatocyte growth factor/scatter factor-mediated cell protection. Oncogene.

[36] Wang R., Kobayashi R., Bishop J.M. (1996). Cellular adherence elicits ligand-independent activation of the Met cell-surface receptor. Proc. Natl. Acad. Sci. USA.

[37] Nakamura Y., Matsubara D., Goto A., Ota S., Sachiko O., Ishikawa S., Aburatani H., Miyazawa K., Fukayama M., Niki T. (2008). Constitutive activation of c-Met is correlated with c-Met overexpression and dependent on cell-matrix adhesion in lung adenocarcinoma cell lines. Cancer Sci..

[38] Mitra A., Sawada K., Tiwari P., Mui K., Gwin K., Lengyel E. (2011). Ligand independent activation of c-Met by fibronectin and α5 β1—Integrin regulates ovarian cancer invasion and metastasis. Oncogene.

[39] Hui A.Y., Meens J.A., Schick C., Organ S.L., Qiao H., Tremblay E.A., Schaeffer E., Uniyal S., Chan B.M.C., Elliott B.E. (2009). Src and FAK mediate cell-matrix adhesion-dependent activation of Met during transformation of breast epithelial cells. J. Cell. Biochem..

[40] Sulzmaier F.J., Jean C., Schlaepfer D.D. (2014). FAK in cancer: Mechanistic findings and clinical applications. Nat. Rev. Cancer.

[41] Basile J.R., Afkhami T., Gutkind J.S. (2005). Semaphorin 4D/plexin-B1 induces endothelial cell migration through the activation of PYK2, Src, and the phosphatidylinositol 3-kinase-Akt pathway. Mol. Cell. Biol..

[42] Giordano S., Corso S., Conrotto P., Artigiani S., Gilestro G., Barberis D., Tamagnone L., Comoglio P.M. (2002). The semaphorin 4D receptor controls invasive growth by coupling with Met. Nat. Cell Biol..

[43] Basile J.R., Castilho R.M., Williams V.P., Gutkind J.S. (2006). Semaphorin 4D provides a link between axon guidance processes and tumor-induced angiogenesis. Proc. Natl. Acad. Sci. USA.

[44] Conrotto P., Corso S., Gamberini S., Comoglio P.M., Giordano S. (2004). Interplay between scatter factor receptors and B plexins controls invasive growth. Oncogene.

[45] Rody A., Holtrich U., Gaetje R., Gehrmann M., Engels K., von Minckwitz G., Loibl S., Diallo-Danebrock R., Ruckhäberle E., Metzler D. (2007). Poor outcome in estrogen receptor-positive breast cancers predicted by loss of plexin B1. Clin. Cancer Res..

[46] Rody A., Karn T., Ruckhäberle E., Hanker L., Metzler D., Müller V., Solbach C., Ahr A., Gätje R., Holtrich U. (2009). Loss of Plexin B1 is highly prognostic in low proliferating ER positive breast cancers—Results of a large scale microarray analysis. Eur. J. Cancer.

[47] Soong J., Chen Y., Shustef E.M., Scott G.A. (2012). Sema4D, the ligand for Plexin B1, suppresses c-Met activation and migration and promotes melanocyte survival and growth. J. Investig. Dermatol..

[48] Peschard P., Fournier T.M., Lamorte L., Naujokas M.A., Band H., Langdon W.Y., Park M. (2001). Mutation of the c-Cbl TKB domain binding site on the Met receptor tyrosine kinase converts it into a transforming protein. Mol. Cell.

[49] Kermorgant S., Parker J. (2005). c-MET signalling: Spatio-temporal decisions. Cell Cycle.

[50] Parachoniak C.A., Luo Y., Abella J.V., Keen J.H., Park M. (2011). GGA3 functions as a switch to promote Met receptor recycling, essential for sustained ERK and cell migration. Dev. Cell.

[51] Hammond D.E., Urbe S., vande Woude G.F., Clague M.J. (2001). Down-regulation of MET, the receptor for hepatocyte growth factor. Oncogene.

[52] Ceresa B.P., Kao A.W., Santeler S.R., Pessin J.E. (1998). Inhibition of clathrin-mediated endocytosis selectively attenuates specific insulin receptor signal transduction pathways. Mol. Cell. Biol..

[53] Kermorgant S., Parker P.J. (2008). Receptor trafficking controls weak signal delivery: A strategy used by c-Met for STAT3 nuclear accumulation. J. Cell Biol..

[54] Ménard L., Parker P.J., Kermorgant S. (2014). Receptor tyrosine kinase c-Met controls the cytoskeleton from different endosomes via different pathways. Nat. Commun..

[55] Foveau B., Ancot F., Leroy C., Petrelli A., Reiss K., Vingtdeux V., Giordano S., Fafeur V., Tulasne D. (2009). Down-regulation of the Met receptor tyrosine kinase by presenilin-dependent regulated intramembrane proteolysis. Mol. Biol. Cell.

[56] Kopitz C., Gerg M., Bandapalli O.R., Ister D., Pennington C.J., Hauser S., Flechsig C., Krell H.-W., Antolovic D., Brew K. (2007). Tissue inhibitor of metalloproteinases-1 promotes liver metastasis by induction of hepatocyte growth factor signaling. Cancer Res..

[57] Krishnaswamy S., Kanteti R., Duke-cohan J.S., Loganathan S., Liu W., Ma P.C., Sattler M., Singleton P.A., Ramnath N., Innocenti F. (2009). Ethnic differences and functional analysis of MET mutations in lung cancer. Clin. Cancer Res..

[58] Ma P.C., Kijima T., Maulik G., Fox E.A., Sattler M., Griffin J.D., Johnson B.E., Salgia R. (2003). c-MET mutational analysis in small cell lung cancer: Novel juxtamembrane domain mutations regulating cytoskeletal functions. Cancer Res..

[59] Jagadeeswaran R., Jagadeeswaran S., Bindokas V.P., Salgia R. (2007). Activation of HGF/c-Met pathway contributes to the reactive oxygen species generation and motility of small cell lung cancer cells. Am. J. Physiol. Lung Cell. Mol. Physiol..

[60] Tyner J.W., Fletcher L.B., Wang E.Q., Yang W.F., Rutenberg-Schoenberg M.L., Beadling C., Mori M., Heinrich M.C., Deininger M.W., Druker B.J. (2010). MET receptor sequence variants R970C and T992I lack transforming capacity. Cancer Res..

[61] Lee J., Han S., Cho H., Jennings B., Gerrard B., Dean M., Schmidt L., Zbar B., vande Woude G.F. (2000). A novel germ line juxtamembrane Met mutation in human gastric cancer. Oncogene.

[62] Kong-Beltran M., Seshagiri S., Zha J., Zhu W., Bhawe K., Mendoza N., Holcomb T., Pujara K., Stinson J., Fu L. (2006). Somatic mutations lead to an oncogenic deletion of met in lung cancer. Cancer Res..

[63] Timofeevski S.L., McTigue M.A., Ryan K., Cui J., Zou H.Y., Zhu J.X., Chau F., Alton G., Karlicek S., Christensen J.G. (2009). Enzymatic characterization of c-Met receptor tyrosine kinase oncogenic mutants and kinetic studies with aminopyridine and triazolopyrazine inhibitors. Biochemistry.

[64] Jeffers M., Schmidt L., Nakaigawa N., Webb C.P., Weirich G., Kishida T., Zbar B., vande woude G.F. (1997). Activating mutations for the Met tyrosine kinase receptor in human cancer. Proc. Natl. Acad. Sci. USA.

[65] Jeffers M., vande Woude G.F. (1999). Activating mutations in the Met receptor overcome the requirement for autophosphorylation of tyrosines crucial for wild type signaling. Oncogene.

[66] Chiara F., Michieli P., Pugliese L., Comoglio P.M. (2003). Mutations in the met oncogene unveil a “dual switch” mechanism controlling tyrosine kinase activity. J. Biol. Chem..

[67] Chen H.-J., Mok T.S., Chen Z.-H., Guo A.-L., Zhang X.-C., Su J., Wu Y.-L. (2009). Clinicopathologic and molecular features of epidermal growth factor receptor T790M mutation and c-MET amplification in tyrosine kinase inhibitor-resistant Chinese non-small cell lung cancer. Pathol. Oncol. Res..

[68] Bean J., Brennan C., Shih J.-Y., Riely G., Viale A., Wang L., Chitale D., Motoi N., Szoke J., Broderick S. (2007). MET amplification occurs with or without T790M mutations in EGFR mutant lung tumors with acquired resistance to gefitinib or erlotinib. Proc. Natl. Acad. Sci. USA.

[69] Engelman J.A., Zejnullahu K., Mitsudomi T., Song Y., Hyland C., Park J.O., Lindeman N., Gale C.-M., Zhao X., Christensen J. (2007). MET amplification leads to gefitinib resistance in lung cancer by activating ERBB3 signaling. Science.

[70] Park S., Choi Y., Sung C.O., An J., Seo J., Ahn M., Ahn J.S., Park K., Shin Y.K., Erkin O.C. (2012). High MET copy number and MET overexpression: Poor outcome in non-small cell lung cancer patients. Histol. Histopathol..

[71] Sun W., Song L., Ai T., Zhang Y., Gao Y., Cui J. (2013). Prognostic value of MET, cyclin D1 and MET gene copy number in non-small cell lung cancer. J. Biomed. Res..

[72] Preusser M., Streubel B., Berghoff A.S., Hainfellner J.A., von Deimling A., Widhalm G., Dieckmann K., Wöhrer A., Hackl M., Zielinski C. (2014). Amplification and overexpression of CMET is a common event in brain metastases of non-small cell lung cancer. Histopathology.

[73] Tsuta K., Kozu Y., Mimae T., Yoshida A., Kohno T., Sekine I., Tamura T., Asamura H., Furuta K., Tsuda H. (2012). c-MET/phospho-MET protein expression and MET gene copy number in non-small cell lung carcinomas. J. Thorac. Oncol..

[74] Elia G., Ren Y., Lorenzoni P., Zarnegar R., Burger M.M., Rusciano D. (2001). Mechanisms regulating c-met overexpression in liver-metastatic B16-LS9 melanoma cells. J. Cell. Biochem..

[75] Ogunwobi O.O., Puszyk W., Dong H.J., Liu C. (2013). Epigenetic upregulation of HGF and c-Met drives metastasis in hepatocellular carcinoma. PLoS ONE.

[76] Garofalo M., Romano G., di Leva G., Nuovo G., Jeon Y., Ngankeu A., Sun J., Lovat F., Alder H., Condorelli G. (2011). EGFR and MET receptor tyrosine kinase—Altered microRNA expression induces tumorigenesis and gefitinib resistance in lung cancers. Nat. Med..

[77] Rahimi N., Tremblay E., Mcadam L., Park M., Schwall R., Eliiott B., Kl O., Canada N.R., Victoria R. (1996). Identification of a hepatocyte growth factor autocrine loop in a murine mammary carcinoma. Cell Growth Differ..

[78] Trovato M., Vitarelli E., Grosso M., Alesci S., Benvenga S., Trimarchi F., Barresi G. (2004). Immunohistochemical expression of HGF, c-MET and transcription factor STAT3 in colorectal tumors. Eur. J. Histochem..

[79] Masuya D., Huang C., Liu D., Nakashima T., Kameyama K., Haba R., Ueno M., Yokomise H. (2004). The tumour-stromal interaction between intratumoral c-Met and stromal hepatocyte growth factor associated with tumour growth and prognosis in non-small-cell lung cancer patients. Br. J. Cancer.

[80] Yano S., Yamada T., Takeuchi S., Tachibana K., Minami Y., Yatabe Y., Mitsudomi T., Tanaka H., Kimura T., Kudoh S. (2011). Hepatocyte growth factor expression in EGFR mutant lung cancer with intrinsic and acquired resistance to tyrosine kinase inhibitors in a Japanese cohort. J. Thorac. Oncol..

[81] Bhardwaj V., Cascone T., Cortez M.A., Amini A., Evans J., Komaki R.U., Heymach J.V., Welsh J.W. (2013). Modulation of c-Met signaling and cellular sensitivity to radiation: Potential implications for therapy. Cancer.

[82] Qian L.-W., Mizumoto K., Inadome N., Nagai E., Sato N., Matsumoto K., Nakamura T., Tanaka M. (2003). Radiation stimulates HGF receptor/c-Met expression that leads to amplifying cellular response to HGF stimulation via upregulated receptor tyrosine phosphorylation and MAP kinase activity in pancreatic cancer cells. Int. J. Cancer.

[83] De Bacco F., Luraghi P., Medico E., Reato G., Girolami F., Perera T., Gabriele P., Comoglio P.M., Boccaccio C. (2011). Induction of MET by ionizing radiation and its role in radioresistance and invasive growth of cancer. J. Natl. Cancer Inst..

[84] Barcellos-Hoff M.H., Park C., Wright E.G. (2005). Radiation and the microenvironment—Tumorigenesis and therapy. Nat. Rev. Cancer.

[85] Thiery J.P., Sleeman J.P. (2006). Complex networks orchestrate epithelial-mesenchymal transitions. Nat. Rev. Mol. Cell Biol..

[86] Kavanagh J.N., Redmond K.M., Schettino G., Prise K.M. (2013). DNA double strand break repair: A radiation perspective. Antioxid. Redox Signal..

[87] Medová M., Aebersold D.M., Zimmer Y. (2012). MET inhibition in tumor cells by PHA665752 impairs homologous recombination repair of DNA double strand breaks. Int. J. Cancer.

[88] Sheng-Hua C., Yan-Bin M., Zhi-An Z., Hong Z., Dong-Fu F., Zhi-Qiang L., Xian-Hou Y. (2007). Radiation-enhanced hepatocyte growth factor secretion in malignant glioma cell lines. Surg. Neurol..

[89] Delitto D., Vertes-George E., Hughes S.J., Behrns K.E., Trevino J.G. (2014). c-Met signaling in the development of tumorigenesis and chemoresistance: Potential applications in pancreatic cancer. World J. Gastroenterol..

[90] Shah A.N., Summy J.M., Zhang J., Park S.I., Parikh N.U., Gallick G.E. (2007). Development and characterization of gemcitabine-resistant pancreatic tumor cells. Ann. Surg. Oncol..

[91] Tang M.K.S., Zhou H.Y., Yam J.W.P., Wong A.S.T. (2010). c-Met overexpression contributes to the acquired apoptotic resistance of nonadherent ovarian cancer cells through a cross talk mediated by phosphatidylinositol 3-kinase and extracellular signal-regulated kinase 1/2. Neoplasia.

[92] Chen J.-T., Huang C.-Y., Chiang Y.-Y., Chen W.-H., Chiou S.-H., Chen C.-Y., Chow K.-C. (2008). HGF increases cisplatin resistance via down-regulation of AIF in lung cancer cells. Am. J. Respir. Cell Mol. Biol..

[93] Marchion D.C., Bicaku E., Xiong Y., Zgheib N.B., al Sawah E., Stickles X.B., Judson P.L., Lopez A.S., Cubitt C.L., Gonzalez-Bosquet J. (2013). A novel c-Met inhibitor, MK8033, synergizes with carboplatin plus paclitaxel to inhibit ovarian cancer cell growth. Oncol. Rep..

[94] Yashiro M., Nishii T., Hasegawa T., Matsuzaki T., Morisaki T., Fukuoka T., Hirakawa K. (2013). A c-Met inhibitor increases the chemosensitivity of cancer stem cells to the irinotecan in gastric carcinoma. Br. J. Cancer.

[95] Ide T., Kitajima Y., Miyoshi A., Ohtsuka T., Mitsuno M., Ohtaka K., Koga Y., Miyazaki K. (2006). Tumor-stromal cell interaction under hypoxia increases the invasiveness of pancreatic cancer cells through the hepatocyte growth factor/c-Met pathway. Int. J. Cancer.

[96] Pennacchietti S., Michieli P., Galluzzo M., Mazzone M., Giordano S., Comoglio P.M. (2003). Hypoxia promotes invasive growth by transcriptional activation of the met protooncogene. Cancer Cell.

[97] Taron M., Ichinose Y., Rosell R., Mok T., Massuti B., Zamora L., Mate J.L., Manegold C., Ono M., Queralt C. (2005). Activating mutations in the tyrosine kinase domain of the epidermal growth factor receptor are associated with improved survival in gefitinib-treated chemorefractory lung adenocarcinomas. Clin. Cancer Res..

[98] Gazdar A.F. (2009). Activating and resistance mutations of EGFR in non-small-cell lung cancer: Role in clinical response to EGFR tyrosine kinase inhibitors. Oncogene.

[99] Benedettini E., Sholl L.M., Peyton M., Reilly J., Ware C., Davis L., Vena N., Bailey D., Yeap B.Y., Fiorentino M. (2010). Met activation in non-small cell lung cancer is associated with de novo resistance to EGFR inhibitors and the development of brain metastasis. Am. J. Pathol..

[100] Cappuzzo F., Jänne P.A., Skokan M., Finocchiaro G., Rossi E., Ligorio C., Zucali P.A., Terracciano L., Toschi L., Roncalli M. (2009). MET increased gene copy number and primary resistance to gefitinib therapy in non-small-cell lung cancer patients. Ann. Oncol..

[101] Cappuzzo F., Marchetti A., Skokan M., Rossi E., Gajapathy S., Felicioni L., del Grammastro M., Sciarrotta M.G., Buttitta F., Incarbone M. (2009). Increased MET gene copy number negatively affects survival of surgically resected non-small-cell lung cancer patients. J. Clin. Oncol..

[102] Nakagawa T., Takeuchi S., Yamada T., Nanjo S., Ishikawa D., Sano T., Kita K., Nakamura T., Matsumoto K., Suda K. (2012). Combined therapy with mutant-selective EGFR inhibitor and Met kinase inhibitor for overcoming erlotinib resistance in EGFR-mutant lung cancer. Mol. Cancer Ther..

[103] Chen X., Zhou J.-Y., Zhao J., Chen J.-J., Ma S.-N., Zhou J.-Y. (2013). Crizotinib overcomes hepatocyte growth factor-mediated resistance to gefitinib in EGFR-mutant non-small-cell lung cancer cells. Anticancer Drugs.

[104] Rho J.K., Choi Y.J., Kim S.Y., Kim T.W., Choi E.K., Yoon S.-J., Park B.M., Park E., Bae J.H., Choi C.-M. (2014). MET and AXL inhibitor NPS-1034 exerts efficacy against lung cancer cells resistant to to EGFR kinase inhibitors due to MET or AXL activation. Cancer Res..

[105] Huang L., An S.J., Chen Z.H., Su J., Yan H.H., Wu Y.L. (2014). MET expression plays differing roles in non-small-cell lung cancer patients with or without EGFR mutation. J. Thorac. Oncol..

[106] Dziadziuszko R., Wynes M.W., Singh S., Asuncion B.R., Ranger-Moore J., Konopa K., Rzyman W., Szostakiewicz B., Jassem J., Hirsch F.R. (2012). Correlation between MET gene copy number by silver in situ hybridization and protein expression by immunohistochemistry in non-small-cell lung cancer. J. Thorac. Oncol..

[107] Etnyre D., Stone A.L., Fong J.T., Jacobs R.J., Uppada S.B., Botting G.M., Rajanna S., Moravec D.N., Shambannagari M.R., Crees Z. (2014). Targeting c-Met in melanoma. Cancer Biol. Ther..

